# A Review of Ambient Air Pollution as a Risk Factor for Posterior Segment Ocular Diseases

**DOI:** 10.3390/jcm12113842

**Published:** 2023-06-04

**Authors:** Agne Markeviciute, Jessie Huang-Lung, Reda Zemaitiene, Andrzej Grzybowski

**Affiliations:** 1 Department of Ophthalmology, Medical Academy, Lithuanian University of Health Sciences, 44307 Kaunas, Lithuania; 2 School of Optometry and Vision Science, University of New South Wales, Sydney 2052, Australia; 3 Institute for Research in Ophthalmology, Foundation for Ophthalmology Development, 61-553 Poznan, Poland

**Keywords:** ambient air pollutants, air pollution, eye disorders, eye diseases, glaucoma, age related macular degeneration, retinal vascular diseases

## Abstract

Purpose. To review the most recent evidence on the association of ambient air pollution with posterior segment ocular diseases. Methods. A search of the most recently published medical literature was performed in PubMed and Google Scholar on 10 December 2022. Articles published between 2018 and December 2022 were included in this rapid review. Studies that evaluated the association between ambient air pollutants (nitrogen dioxide (NO_2_), carbon monoxide (CO), sulfur dioxide (SO_2_), ozone (O_3_), particulate matters (PM_s_), total hydrocarbons (THC), nonmethane hydrocarbons (NMHC), benzene), and ocular posterior segment diseases (glaucoma, age-related macular degeneration (AMD), and retinal vascular diseases) were included. Results. Nineteen research articles met the inclusion criteria. Significant associations were found between PM_2.5_ and glaucoma, including primary open angle, primary angle closure, and normal tension glaucoma. An increased risk of AMD was linked to increased exposure to PM_2.5_, NO_2_, and CO. Single studies suggested that increased exposure to PM_2.5_ and PM_10_ is associated with diabetic retinopathy; THC and NMHC increased the risk of retinal vein occlusion; and CO, NO_2_, and PM_10_ are linked to an increased risk of central retinal artery occlusion. Conclusions. There is increasing evidence that toxic air pollutants have an impact on posterior segment ocular diseases, hence determining it as a potential modifiable risk factor for visual impairment.

## 1. Introduction

Air pollution is responsible for approximately 6.7 million premature deaths per year globally [1]. It is linked to non-communicable diseases including cardiovascular diseases, stroke, chronic obstructive pulmonary disease, and lung cancer [2], and is also associated with increased risk of autoimmune and intestinal diseases [3,4].

In the past 20 years, an upward trend in ambient air pollution has been observed. Air pollutants are generated as by-products of industrial processes (e.g., mining, manufacturing, building), motor vehicle fuel combustion, household burning of solid fuel, and from natural sources such as bushfires, dust storms, and pollens. Air pollutants include gases, such as nitrogen dioxide (NO_2_), sulfur dioxide (SO_2_), ozone (O_3_), and carbon monoxide (CO), and particulate matter (PM) [5]. Differentiated by size, PM_2.5_ refers to particles with a diameter of ≤2.5 μm and PM_10_ refers to particles with a diameter of ≤10 μm. Both PM_2.5_ and PM_10_ can be inhaled and enter the lungs, while PM_2.5_ is small enough to even enter the bloodstream.

Due to direct contact with the external environment, the eye is one of the most vulnerable organs to airborne pollutants. The impact of air pollution on the ocular surface is well documented [6]. However, there is emerging evidence of air pollutants impacting the posterior segment of the eye. Exposure to air pollutants may affect retinal and choroidal hemodynamics, leading to retinal vessel occlusion [7]; furthermore, it may be associated with an increased risk of glaucoma, and age-related macular degeneration (AMD) [8]. Different air pollutants have variable associations with ocular morbidity, vision impairment, and reduced quality of life.

As air pollution is becoming an increasing global problem, it is important to understand its impact on ocular health and vision impairment to inform planning of public health strategies to prevent ocular morbidity. Our aim is to review and update recent evidence of air pollution’s effect on posterior segment ocular diseases, specifically the role of air pollution as a potential risk factor for AMD, glaucoma, and retinal vascular diseases (diabetic retinopathy and retinal vascular occlusion).

## 2. Materials and Methods

A search of the medical literature was performed in PubMed and Google Scholar. The keywords, terms, and medical subject headings (MeSH) searched for are listed in Table 1. Emphasis was placed on the most recent articles published from 2018 up to 10 December 2022. This work builds upon the recent reviews conducted by Lin et al. [8] and Grant et al. [9]. This review focuses on the most recent published data and includes studies on retinal vascular diseases that were not covered in previous reviews.

Original research articles published in English were eligible for inclusion. Further inclusion criteria included studies that evaluated the association between ambient air pollutants (nitrogen dioxide (NO_2_), carbon monoxide (CO), sulfur dioxide (SO_2_), ozone (O_3_), particulate matters (PM_s_), total hydrocarbons (THC), nonmethane hydrocarbons (NMHC), benzene), and ocular posterior segment diseases: glaucoma, AMD, and retinal vascular diseases. We included studies that used longitudinal, prospective, or retrospective cohort, repeated-measures panel, cross-sectional case-control and case-crossover, experimental designs. Nineteen research articles met the inclusion criteria.

## 3. Results

The summary of the studies and different air pollutants’ toxic effects are presented in Table 2 and Table 3.

## 4. Glaucoma

Age, family history, ethnicity, and high intraocular pressure (IOP) are well-established risk factors for glaucoma; however, the impact of air pollution is not currently well understood. Reports that the risk of glaucoma is 1.5 times higher for people living in urban areas than those living in rural areas [28] have led to the hypothesis that air pollution is a potential, modifiable risk factor for glaucoma [28].

A case-control study that compared 645 people with incident open angle glaucoma and 2580 people without glaucoma (controls) over 5 years found that PM_2.5_ exposure was an independent risk factor for primary open angle glaucoma (POAG) [12]. Exposure was based on atmospheric PM_2.5_ concentration according to air quality monitoring data at the participant’s residential address [12].

Multivariable logistic regression models showed increased odds (odds ratio (OR):1.668) of developing POAG in the group exposed to WHO 2.0 Level (≥2 × 25 mg/m^3^ × exposure months) compared to the normal level (<25 mg/m^3^ × exposure months) [12]. A similar finding was found when the analysis was restricted to people with diabetes, with the odds of developing glaucoma in those exposed to the highest quartile of atmospheric PM_2.5_ concentrations being 1.7× higher than those in the lowest quartile [13].

The odds of self-reported glaucoma increased by 6% for each interquartile range increase in PM_2.5_, although the PM_2.5_ concentration in the analysis by Chua and colleagues was within the WHO ambient air quality values of annual means of 10 μg/m^3^ [10]. Increased odds of self-reported glaucoma were associated with greater exposure to ambient PM_2.5_ (OR = 1.06) [10]. Furthermore, exposure to higher levels of PM_2.5_ was associated with a thinner macular ganglion cell–inner plexiform layer measured with spectral domain optical coherence tomography (SD-OCT) [10]. No significant relationship between PM_2.5_ concentration and intraocular pressure was found [10].

Results from a study on aging by Grant et al. complement previous findings [15]. The risk of self-reported glaucoma increased with the increase in PM_2.5_ levels (OR = 1.14) [15]. The association became even stronger after including multiple pollutants (O_3_, SO_2_, NO_2_) in the analysis (OR = 1.24). Furthermore, in the multi-pollutant model, IOP was significantly associated with increased levels of O_3_ and PM_2.5_, while the single pollutant model did not show an association between PM_2.5_ and IOP [15].

Few studies evaluated the effect of PM_2.5_ on subtypes of glaucoma.

The results of a long-term nationwide cross-sectional study conducted in China showed that each 10 μg/m^3^ increment in PM_2.5_ increased the odds of primary glaucoma (OR = 1.07) and angle-closure glaucoma (PACG) (OR = 1.14) [14]. Estimated levels of PM_2.5_ during the 17-year study period ranged from 28.0 to 96.4 μg/m3 with an average of 62.4 μg/m^3^ [14]. In the subgroup analyses the odds of PACG increased by 14% for each 10 μg/m3 increment in PM_2.5_, while a positive but non-significant association was found between PM_2.5_ exposure and POAG [14]. The authors hypothesized that PACG may be more sensitive to ambient PM_2.5_ exposure [14]. No significant association was revealed between increased long-term PM_2.5_ exposure and IOP [14].

The data of 1006 patients with normal tension glaucoma (NTG) and 2533 patients with POAG from Taiwan’s Longitudinal Health Insurance Database were included in a case-control study [17]. The risk of NTG increased significantly as exposure to PM_2.5_ increased when the estimated average PM_2.5_ level was 1366.8 μg/m^3^ [17]. The association between PM_2.5_ exposure and NTG was concentration-dependent; multiple logistic regression showed that the risk of NTG increased more as PM_2.5_ levels increased when compared to the risk of POAG [17].

Children are no exception when it comes to the evaluation of harmful air pollution’s effects. The development of childhood glaucoma was found to be associated with exposure to higher levels of PM_10_ in a study by Min et al. [11]. A longitudinal study included 9004 infants born in 2002 who were followed over 11 years. Childhood glaucoma was diagnosed in 85 children (0.94%) and was significantly associated with increased levels of PM_10_ [11].

It was observed that ambient air pollution can contribute to an increased number of outpatient visits for glaucoma. The results of a study by Wang et al. showed that each 10-unit increase in the exposure of further pollutants was associated with an increased number of outpatient visits for primary glaucoma with the following excess risk (ER) values: PM_10_ (0.7%), SO_2_ (2.4%), and NO_2_ (4.1%) [16]. The results showed that SO_2_ and NO_2_ were associated with greater risk [16].

Similarly, the odds of an outpatient visit for acute glaucoma (including acute angle closure glaucoma and glaucomatocyclitic crisis) significantly increased with the increase in exposure to PM_2.5_ (OR = 1.07), NO_2_ (OR = 1.12) per IQR increase, over a lag of 0–3 days, which showed higher and more sustained results than PM_10_ (OR = 1.03) and CO (OR = 1.04) over a lag of 0–1 days [18]. Therefore, it can be assumed that air pollutants are significantly associated with an increased risk of acute glaucoma [18].

## 5. Age-Related Macular Degeneration

The etiology and pathogenesis of AMD have been linked to the interaction of genetic and external factors, such as smoking [21,29]. However, the exact etiology of AMD has not been fully elucidated to date. The key pathways to AMD development are likely associated with excessive oxidative stress and persistent inflammation in the central retina [21]. The results of studies revealed that exposure to air pollutants may be associated with the induction of oxidative stress and inflammation; thus, it may play a significant role in the development of AMD [21].

Certain air pollutants’ effect on early AMD, late AMD, and any AMD (the presence of early or late AMD) was explored in a study by Ju et al. by comparing data from 14,021 subjects without AMD and 1094 subjects with AMD [21]. The odds of early AMD increased with increased exposure to NO_2_ (OR = 1.24), CO (OR = 1.22), and PM_10_ (OR = 1.13) in the fully adjusted models [21]. Similar results were found for any AMD [21]. Furthermore, a significantly higher prevalence of early AMD in the current year was found in participants exposed to high levels of PM_10_ (>50 μg/m^3^), NO_2_ (>30 ppb), and CO (>500 ppb) compared to participants who were exposed to low levels of pollutants, while O_3_ (>24 ppb) in the current year was associated with a significantly lower prevalence of early AMD [21]. No significant association between the exposure to high levels of PM_10_, SO_2_, NO_2_, and O_3_ and late AMD was found [21].

Multiple logistic regression models were used to quantify the association of environmental variables with any AMD versus non-AMD patients, any exudative AMD versus nonexudative AMD, and active exudative AMD versus inactive exudative and nonexudative AMD [23]. The results showed that O_3_ and NO_2_ significantly increased the risk of active exudative AMD (ORs: 1.014, 1.005, respectively), while O_3_ was significantly associated with any AMD when compared with non-AMD patients (OR = 1.011) [23].

Increased risk of AMD was linked to chronic exposure to increased levels of ambient NO_2_ and CO [19]. Results were obtained from a study that included data from 39,819 subjects from a nationwide dataset between the years 2000 and 2010 [19]. Air pollutant concentrations were grouped into four levels based on quartile: NO_2_ concentration (Q1: <6563.2, Q2: 6563.2–8238.2, Q3: 8238.3–9825.5, and Q4: >9825.5 ppb) and CO concentration (Q1: <195.7, Q2: 195.7–241.7, Q3: 241.8–297.1, and Q4: >297.1 ppm) [19]. The highest NO_2_ exposure (Q4) significantly increased AMD incidence from 25.60 to 51.52 per 10,000 person-years when compared with the lowest exposure (Q1) (the adjusted HR = 1.91 for the highest NO_2_ quartile) [19]. Similarly, the Q4 CO level increased the incidence from 28.89 to 56.24 (adjusted HR = 1.84 for Q4) [19]. However, the dose–response effect was not confirmed [19].

The association between self-reported AMD and a wide range of pollutants including PM_2.5_, PM_2.5_ absorbance, PM_10_, NO_2_, and NOx was assessed in a study by Chua and colleagues [20]. They used SD-OCT to evaluate changes in the thickness of retinal layers [20]. The authors analyzed data from 52,602 participants in UK Biobank and measured photoreceptor sublayer thickness and retinal pigment epithelium (RPE) layer thickness [20]. The results showed that higher odds of self-reported AMD (OR = 1.08), a thinner photoreceptor synaptic region, a thicker photoreceptor inner segment layer, and a thinner RPE were significantly associated with increased exposure to PM_2.5_ [20].

Grant et al. performed a large, longitudinal study on aging to evaluate the association between PM_2.5_ and AMD [15]. They defined visual impairment as binocular acuity worse than 20/40 (0.301 logMAR), and late-stage AMD was distinguished from early AMD and determined as self-reported AMD with visual impairment. A significant association was found between visual impairment (OR = 1.12), visually impairing AMD (OR = 1.51), and people living in areas with higher mean levels of PM_2.5_ in a single pollutant model [15]. No significant association was found between higher levels of PM_2.5_ and AMD with no visual impairment [15]. The authors hypothesized that the possible cause of the weak relationship between PM_2.5_ and AMD may be that PM_2.5_ does not affect the early AMD process when visual acuity is not reduced [15].

A significant relationship between chronic exposure to PM_2.5_ and increased risk of AMD was revealed by the results of a large-scale longitudinal study that evaluated data from 4,284,128 participants in Taiwan between the years 2001 and 2011 [22]. The levels of PM_2.5_ were continuously measured by satellites and the estimated annual mean of PM_2.5_ exposure was 34.23 ± 7.17 μg/m^3^ [22]. The distributed lag nonlinear model showed that the risk of AMD increased by 19% for a 10 μg/m^3^ PM_2.5_ increase [22].

## 6. Retinal Vascular Diseases

Air pollutant exposure can increase the risk of vascular conditions, including both macrovascular and microvascular conditions.

The higher risk of diabetic retinopathy (DR) in patients with diabetes mellitus was reported to be associated with higher PM exposure [24]. These results were submitted by Pan et al. who performed a retrospective study including data from 579 patients with DR and 5790 patients with no DR (controls) [24]. The mean PM_2.5_ exposure level was 33.4 μg/m^3^ and that of PM_2.5–10_ was 25.8 μg/m^3^. The odds of DR occurrence increased with every 10-μg/m^3^ increase in PM_2.5_ (OR = 1.29) and PM_2.5–10_ (OR = 1.37) [24]. Similar results with higher odds were obtained in a study by Shan and colleagues [25]. Each 10 μg/m^3^ increase in PM_2.5_ increased the risk of DR in patients with DM (OR = 1.41) [25]. Both of these studies provide evidence for a significant association between higher levels of PM and increased risk of DR.

Long-term exposure to total hydrocarbons (THCs) and nonmethane hydrocarbons (NMHCs) can increase the risk of retinal vein occlusion (RVO) [26]. Data from 855,297 patients were included in a retrospective study to assess the impact of pollutants on the risk of developing RVO over five years [26]. Newly diagnosed RVO was significantly associated with increased exposure to THC (HR = 19.88 at 0.51-ppm increases in THC) and NMHC (HR = 4.33 at 0.27-ppm increases in NMHC) in a non-adjusted single pollutant model [26]. The dose–response relationship was revealed for these two pollutants [26].

The association between central retinal artery occlusion (CRAO) and the levels of PM _2.5_, PM _10_, benzene, CO, NO_2_, SO_2_, and O_3_ was evaluated by Grzybowski et al. [27]. A total of 2272 cases of newly diagnosed CRAO were included in a retrospective study [27]. Single-factor regression analysis showed that CRAO occurrence was significantly associated with concentrations (measured on the day of CRAO occurrence) of CO (incidence rate ratio (IRR) = 1.29), NO_2_ (IRR = 1.02), and PM_10_ (IRR = 1.01) [27]. Additionally, they reported a positive association between short-term daily changes in PM_10_, NO_2_, SO_2_, O_3_, and CO concentrations, as well as with air temperature in the days preceding the diagnosis of CRAO [27].

## 7. Discussion

There is a limited number of studies evaluating the association between air pollution and ocular diseases as this research requires long-term evaluation and large sample sizes. Different studies included various pollutants and evaluated different parameters, thus making the comparison and generalized conclusions challenging.

Ambient PM_2.5_ is reported to be the fifth-ranked risk factor of mortality globally [14]; furthermore, it may play a significant role in the development of vision-threatening posterior segment ocular diseases. Our reviewed studies showed that increased exposure to PM_2.5_ is linked to the increased incidence of glaucoma, suggesting that this pollutant is an independent risk factor for the disease [9,10,12,14,15,17]. Several studies indicated significant primary findings of possible associations between exposure to PM_2.5_ and increased incidence of subtypes of glaucoma, including PACG [14], NTG [17], and acute glaucoma [18]. Evidence of the association between increased exposure to PM_2.5_ and glaucoma was verified by the results of a recently published systematic review and meta-analysis by Grant and colleagues [9].

Grant et al. found that IOP was significantly associated with increased levels of O_3_ and PM_2.5_ in the multi-pollutant model, suggesting increased toxic effects by correlation of different pollutants [15]. More evidence of the pathogenic mechanisms of air pollutants’ effect on IOP arises from animal and in vitro studies [30]. IOP increased gradually after mouse eyes were exposed to topical PM_2.5_ suspension for 3 months [30]. Human trabecular meshwork (HTM) cells subjected to various PM_2.5_ concentrations in vitro resulted in decreased cell viability (concentration-dependent decrease) and affected its contraction, increased oxidative stress, and increased the subsequent induction of NLRP3 inflammasome-mediated pyroptosis [30]. This may damage the trabecular meshwork, leading to an increased aqueous outflow resistance, thereby causing IOP elevation and glaucoma [30]. Furthermore, black carbon was significantly linked to an increased IOP when the oxidative stress allelic score was increased [31]. The authors suggested that susceptibility to black carbon exposure might be determined biologically and there is a possibility of gene–environment interactions [31]. There is increasing evidence that oxidative stress could be one of the possible pathogenetic mechanisms in the association between air pollution and IOP [30,31]. To the contrary, Chua et al. proposed neurotoxicity or vascular dysfunction as possible pathogenetic mechanisms as they did not find a significant relationship between PM_2.5_ and IOP [10].

It was noticed that patients with comorbidities such as diabetes might have a higher risk of glaucoma when exposure to PM_2.5_ is increased [13]. Likewise, children may be more susceptible to air pollution, as Min et al. found that increased exposure to PM_10_ is associated with an increased risk of childhood glaucoma [11]. Furthermore, increased exposure to air pollutants may affect retinal vascular changes in children. Korsiak et al. found that the combined oxidant capacity of O_3_ and NO_2_ (Ox) was associated with the reduction in retinal arteriolar diameter, while PM_2.5_ was linked to a slight increase in arteriolar diameter [32]. The identified changes in microvasculature can be a significant factor in the further determination of possible pathogenetic mechanisms between air pollution and posterior segment ocular diseases.

Several studies aimed to evaluate ambient air pollutants’ effect on AMD. There has been a lack of consolidated findings on the exact pathogenetic mechanisms between air pollutants and AMD. Increased levels of PM_10_, NO_2_, and CO were found to be associated with early AMD [21], while PM_2.5_ was more related to late AMD and visual impairment [15]. Similarly, the risk of AMD increased with the increased levels of PM_2.5_ [22]. A study by Chua et al. confirmed that greater exposure to PM_2.5_ was associated with self-reported AMD; furthermore, exposure to higher concentrations of PM_2.5_, PM_10_, and PM_2.5_ absorbance were associated with inner and outer retinal layer thinning evaluated by SD-OCT [20], suggesting toxic pollutants’ effect on retinal structures. Ambient PM_2.5_ was reported to be positively associated with pro-angiogenesis molecules; thus, it could induce vascular dysfunction and the AMD neovascularization process, possibly leading to a visual impairment [15].

The results of the evaluation of other pollutants’ effects were not consistent enough; nevertheless, possible associations cannot be denied. Increased levels of NO_2_ were associated with an increased risk of early AMD [21], active exudative AMD [23], and AMD [19]; however, Chua et al. did not find a significant association [20]. The link between the risk of AMD and increased levels of CO was found in two studies [19,21]. NO_2_ is known to be a component of reactive nitrogen species (RNS) that can act together with reactive oxygen species and damage cells, while CO can promote oxidative stress; thus, these pollutants could significantly impact the pathogenesis of AMD [19]. We have observed rising evidence that increased levels of PM_2.5_, NO_2_, and CO could be validated as risk factors for AMD.

AMD can be caused by interactions between genetic and environmental risk factors. It is known that the retina is one of the most metabolically active tissues, making it more susceptible to oxidative stress. The damage increases with age, thus leading to retinal dysfunction and cell loss [29]. As no exact pathogenetic mechanisms between air pollution and AMD have been established, some authors even described a possible link between air pollution and toxicity to retinal ganglion cells and other parts of the retina as “pollution retinopathy” or “particulate pollution-induced retinopathy” [10]. It was reported that air pollution exposure is associated with increased oxidative stress, inflammation, hypercoagulation, and disruption of the blood–brain barrier [33,34]. Specifically, PM-induced changes in endothelial cells and vascular flow were reported to contribute to systemic inflammatory responses through circulating plasma proteins, decreased vascular tone, changes in endothelial cell dynamics, and blood–brain barrier alterations [35].

These mechanisms are known to be associated with AMD as well as with retinal vascular diseases. Single studies showed that increased exposure to PM_2.5_ and PM_2.5–10_ can be associated with an increased risk of DR [24,25]; THC and NMHC can increase the risk of RVO [26], while CO, NO_2_, and PM_10_ were associated with increased risk of CRAO [27]. It is reported that air pollutants can be associated with arterial narrowing, retinal microvascular dysfunction, and inflammatory responses altering endothelial dysfunction, leading to retinal vascular diseases [26,27,36,37].

The exact pathogenetic mechanisms of air pollutants’ effect on posterior segment ocular diseases have not been elucidated to date. Nevertheless, it is believed to be linked to an oxidative stress associated with elevated intracellular levels of reactive oxygen species (ROS) [38]. Excessive ROS formation can lead to vascular endothelial dysfunction and inflammation in the retina [38]. Furthermore, ROS can stimulate the production of inflammatory cytokines, such as tumor necrosis factor-alpha (TNF-α) and interleukin-6 (IL-6), causing inflammation and cell death [38]. Increased levels of ROS have been associated with diabetic retinopathy, glaucoma, and AMD [38].

Laboratory studies by Lee et al. found that PM_2.5_ induced the epithelial–mesenchymal transition (EMT) in retinal pigment epithelial (RPE) cells, which resulted in a changed phenotype of RPE cells from epithelial to fibroblast-like mesenchymal, and increased cell migration [39]. The pathogenetic pathway was activated by cellular ROS production during PM_2.5_-induced EMT which was reported to be implicated in the development of intraocular fibrotic disorders, including AMD, proliferative vitreoretinopathy, and diabetic retinopathy [39].

Although our reviewed studies included large sample sizes, the majority of them are observational. We found only one experimental study and one systematic review and meta-analysis. Different studies used different methodologies, analysis methods, adjustments of confounding variables, and concentrations of air pollutants varied. The epidemiological studies mainly considered the residential address of the participants but did not account for exposure to pollutants at place of employment, where participants may have spent considerable time. The authors of a recent systematic review [9] reported high heterogeneity among the research studies and found a low certainty of their evidence ratings. However, there is an undoubtable association between several air pollutants and posterior segment ocular diseases, including glaucoma, AMD, and retinal vascular diseases, that are significantly associated with vision impairment. There is a need for further longitudinal, comprehensive studies on air pollution’s effects on ocular diseases seeking generalized conclusions and preventative recommendations.

The most common pollutants that were reported to be significantly associated with ocular posterior segment diseases included NO_2_, CO, PM_2.5_, and PM_10_. These are generally considered the most dangerous to various health conditions, as well as for the environment and even mortality. We observed that the majority of our reviewed studies were performed in highly urbanized cities. Urbanization is significantly associated with the increase in air pollution, leading to the previously discussed diseases, increased outpatient visits, and healthcare costs.

There are inherent connections between air pollution, climate change, health issues, and increased costs in different spheres. It was reported in the studies that the most vulnerable to climate-related health problems are children, older people, people in lower socioeconomic groups, and future generations [40]. Significant regulatory changes are urgently needed to effectively control ambient and household air pollution, as well as climate change. It was reported that keeping the air temperature rise below 2°C over the next 50 years would prevent about 4.5 million premature deaths, about 3.5 million hospitalizations and emergency room visits, and approximately 300 million lost workdays in the United States [41]. Policies, technologies, specific emission regulations, and cooperative international efforts have been implemented against air pollutants; however, there is evidence that the results are not effective enough.

## 8. Conclusions

The results of our reviewed studies highlight significant effects of air pollution on ocular posterior segment diseases, possibly leading to irreversible vision impairment. The most common pollutants associated with posterior segment ocular diseases include PM_2.5_, PM_10_, NO_2_, and CO. Furthermore, different pollutants may have additive effects, enhancing toxic effects.

Introducing better technologies and standardized methods to track air pollutants’ levels in the air and taking screening and protection measures should be considered, as these can be cost-effective means to prevent and treat ocular diseases, which are significantly associated with air pollution.

## Figures and Tables

**Table 1:** Keywords, terms, and MeSH searched.

1	Air Pollution
2	ambient air pollutants
3	eye disorders
4	1 and 3; 2 and 3
5	eye diseases
6	1 and 5; 2 and 5
7	glaucoma
8	1 and 7; 2 and 7
9	age related macular degeneration
10	1 and 9, 2 and 9
11	retinal vascular diseases
12	1 and 11; 2 and 11

**Table 2:** Summary of the studies included in this review.

	First Author (Year)	Study Design	Subjects, Dataset, Context	Pollutant(s) Studied	Results	
					Association with the Disease	Additional Findings
Glaucoma	Chua et al. (2019) [10]	Retrospective cohort	111,370 subjects	PM_2.5_	Increased odds of glaucoma in participants residing in areas with higher ambient PM_2.5_ (OR = 1.06 per interquartile range increase)	Higher ambient PM_2.5_ was also associated with thinner macular ganglion cell–inner plexiform layer, a dose–response relationship was observed
			United Kingdom Biobank			
	Min et al. (2019) [11]	Retrospective cohort	9004 infants born in 2002, followed for 11 years	PM_10_	Childhood glaucoma prevalence was 0.94%; increases of 1 μg/m^3^ in long-term PM_10_ were significantly associated with childhood glaucoma (HR = 1.22)	
			National Health Insurance Service database, Republic of Korea			
	Sun et al. (2021) [12]	Case-control	645 incident POAG cases and 2580 controls	PM_2.5_	Exposure to ≥2-fold the WHO normal level (25 mg/m^3^) of ambient PM_2.5_ compared to normal levels was associated with increased odds of developing POAG (OR = 1.668)	
			Longitudinal Health Insurance Database of Taiwan			
	Chiang et al. (2021) [13]	Case-control	197 DM patients with glaucoma and 788 DM patients without glaucoma	PM_2.5_	The odds of glaucoma increased in patients with DM at PM_2.5_ exposure concentration in highest quartile (Q4) compared with in Q1 (OR = 1.731)	
			National Health Insurance Research Database (NHIRD) of Taiwan			
	Yang et al. (2021) [14]	Cross-sectional	33,701 adults from 10 provinces of China	PM_2.5_	The odds of PACG increased by 14% at each 10 μg/m^3^ increment in PM_2.5_	The odds of POAG were positively but not significantly associated with long-term exposure to PM_2.5_ (OR = 1.05)
	Grant et al. (2021) [15]	Cross-sectional	29,147 participants	PM_2.5_, O_3_, SO_2_, NO_2_	Increased odds of glaucoma in subjects living in areas with higher PM_2.5_ levels (OR = 1.14)	PM_2.5_ and O_3_ were associated with increased IOP (multi-pollutant model)
			Canadian Longitudinal Study on Aging			
	Wang et al. (2022) [16]	Multi-model time series analysis	Shenyang	PM_10_ SO_2_, NO_2_, CO, O_3_	The number of outpatient visits for primary glaucoma increases with each 10-unit increase in following exposures: PM_10_ (ER = 0.7%), SO_2_ (ER = 2.4%), NO_2_ (ER = 4.1%); with each 0.1 mg/m^3^ increase in CO (ER = 0.7%.)	The ERs for primary glaucoma in multiple-pollutant models were: NO_2_ + CO (3.6%), NO_2_ + SO_2_ (1.8%), NO_2_ + PM_10_ (0.7%), NO_2_ + CO + PM_10_ (0.7%), NO_2_ + SO_2_ + PM_10_ (0.6%), and NO_2_ + SO_2_ + CO (1.8%)
	Luo et al. (2022) [17]	Case-control	1006 participants in the NTG group and 2533 in the POAG group	PM_2.5_	The risk of developing NTG compared with POAG was more closely related with PM_2.5_ exposure (OR = 1.246 at Q4 level when compared with Q1 level)	PM_2.5_ had a concentration−dose effect
			Taiwan Longitudinal Health Insurance Database 2000 for the 2008 to 2013 period			
	Li et al. (2022) [18]	Case-crossover	14,385 cases of acute glaucoma outpatients Shanghai	PM_2.5_, PM_10_, NO_2_, SO_2_, CO, O_3_	The odds of outpatient visit for acute glaucoma significantly increased with increased exposure to PM_2.5_ (OR = 1.07), PM_10_ (OR = 1.03), NO_2_ (OR = 1.12), and CO (OR = 1.04)	
Age-related macular degeneration	Chang et al. (2019) [19]	Longitudinal population-based study	39,819 subjects	CO, NO_2_	The incidence of AMD increased at the highest NO_2_ exposure (Q4) when compared with Q1 (HR = 1.91); similar for CO (HR = 1.84)	
			Taiwan Longitudinal Health Insurance Database between year 2000 and 2010			
	Grant et al. (2021) [15]	Cross-sectional	29,147 subjects	PM_2.5_, O_3_, SO_2,_ NO_2_	Higher mean levels of PM_2.5_ increased odds of visual impairment (OR = 1.12) and visualy impairing AMD (OR = 1.51)	No significant association was found between higher levels of PM_2.5_ and AMD with no visually impairment
			Canadian Longitudinal Study on Aging			PM_2.5_ correlated with other pollutants such as O_3_, SO_2_, NO_2_
	Chua et al. (2021) [20]	Cross-sectional	52,602 participants	PM_2.5_, PM_2.5_ absorbance, PM_10_, NO_2_ and NOx	The odds of self-reported AMD increased in subjects who were exposed to higher ambient PM_2.5_ levels (OR = 1.08 per IQR increase)	Higher levels of PM_2.5_ were associated with thinner photoreceptor synaptic region, thicker photoreceptor inner segment layer and thinner RPE
						Higher levels of PM_2.5_ absorbance and NO_2_ were associated with thicker photoreceptor inner and outer segment layers, and a thinner RPE layer
			United Kingdom Biobank			Higher levels of PM_10_ were associated with thicker photoreceptor outer segment and thinner RPE
						Higher exposure to NOx was associated with thinner photoreceptor synaptic region
	Ju et al. (2022) [21]	Cross-sectional	15,115 subjects	PM_10_, SO_2_, NO_2_, CO, and O_3_	Significantly higher prevalence of early AMD was found in participants exposed to high levels of PM_10_ (>50 μg/m^3^) at prior years, NO_2_ (>30 ppb) and CO (>500 ppb) at current-to-prior years	Increased levels of O_3_ (>24 ppb) at current-to-prior years were significantly associated with lower prevalence of early AMD
			Korean National Health and Nutrition Examination Survey 2008–2012			
	Liang et al. (2022) [22]	Longitudinal cohort	4,284,128 participants	PM_2.5_	AMD risk increased by 19% for a 10 μg/m3 PM_2.5_ increase	
			Taiwan			
	Hunt et al. (2022) [23]	Database study	9,884,527 subjects	CO, NO_2_, PM_2.5_, O_3_, SO_2_	Increased odds of active exudative AMD were associated with increased levels of O_3_ (OR = 1.014) and NO_2_ (OR = 1.005); any AMD was associated with increased levels of O_3_ (OR = 1.011).	
			Intelligent Research in Sight (IRIS) registry from 2016 to 2018			
			United States			
Retinal vascular diseases	Pan et al. (2019) [24]	Longitudinal cohort	6360 subjects (579 DM cases and 5790 controls)	PM_2.5_, PM_2.5–10_, CO, NO_2_, SO_2_, O_3_	The odds of DR increased for every 10 μg/m^3^ increase in PM_2.5_ (OR = 1.29); in PM_2.5–10_ (OR = 1.37)	
			Longitudinal Health Insurance Database of Taiwan during 2003–2012			
	Shan et al. (2021) [25]	Cross-sectional	3111 participants with DM, 329 of whom were diagnosed with DR	PM_2.5_	The odds of DR increased for each 10 μg/m^3^ increase in PM_2.5_ (OR = 1.41)	
			China			
	Zhang et al. (2019) [26]	Retrospective cohort	855,297 subjects	Total hydrocarbons (THCs) and nonmethane hydrocarbons (NMHCs)	The risk of RVO occurrence increased with increased levels of THC (HR = 19.88 at 0.51 ppm increase) and increased levels of NMHC (HR = 4.33 at 0.27 ppm increase)	
			National Health Institute Research Database			
			Taiwan			
	Grzybowski et al. (2019) [27]	Retrospective, population-based study	2272 subjects	PM_2.5_, PM_10_, benzene, CO, NO_2_, SO_2_, O_3_	CRAO occurrence was significantly associated with increased concentrations of CO (IRR = 1.29), NO_2_ (IRR = 1.02), and PM_10_ (IRR = 1.01)	Positive association between short-term daily changes in PM_10_, NO_2_, SO_2_, O_3_, and CO concentrations in the days preceding the diagnosis of CRAO
			Polish National Health Service database			

THC—total hydrocarbon, NMHC—nonmethane hydrocarbon, NO_2_—nitrogen dioxide, CO—carbon monoxide, SO_2_—sulfur dioxide, O_3_—ozone, PM—particulate matter, IOP—intraocular pressure, Q—quartile, POAG—primary open angle glaucoma, PACG—primary angle closure glaucoma, WHO—world health organization, HTM—human trabecular meshwork, OR—odds ratio, HR—hazard ratio, ER—excess risk, AMD—age-related macular degeneration, DR—diabetic retinopthy, DM—diabetes mellitus, RVO—retinal vein occlusion, CRAO—central retinal artery occlusion, NTG—normal tension glaucoma, IRR—incidence rate ratio.

**Table 3:** Air pollutants’ effect on posterior segment ocular diseases.

Condition	Air Pollutants	References
Primary open-angle glaucoma	PM_2.5_	Chua et al. [10], Sun et al. [12], Chiang et al. [13], Grant et al. [15]
Primary angle-closure glaucoma	PM_2.5_	Yang et al. [14]
Childhood glaucoma	PM_10_	Min et al. [11]
Increased outpatient visits for primary glaucoma	PM_10_ SO_2_, NO_2_, CO	Wang et al. [16]
Normal-tension glaucoma	PM_2.5_	Luo et al. [17]
Increased outpatient visits for acute glaucoma	PM_2.5_, PM_10_, NO_2_, CO	Li et al. [18]
Age-related macular degeneration	NO_2_, CO	Chang et al. [19], Ju et al. [21]
	PM_2.5_	Grant et al. [15], Chua et al. [20], Liang et al. [22]
	PM_10_	Ju et al. [21]
	NO_2_, O_3_	Hunt et al. [23]
Diabetic retinopathy	PM_2.5_, PM_2.5–10_	Pan et al. [24], Shan et al. [25]
Retinal vein occlusion	THC, NMHC	Zhang et al. [26]
Central retinal artery occlusion	CO, NO_2_, PM_10_	Grzybowski et al. [27]

THC—total hydrocarbon, NMHC—nonmethane hydrocarbon, NO_2_—nitrogen dioxide, CO—car-bon monoxide, O_3_—ozone, PM—particulate matter.

## Data Availability

Data supporting the findings of this study are available within the included articles or published studies.
